# Struck by stroke - experiences of living with stroke in a rural area in Uganda

**DOI:** 10.1186/s12889-023-15832-3

**Published:** 2023-06-05

**Authors:** Linda Timm, Julius Kamwesiga, Sulaiman Kigozi, Charlotte Ytterberg, Gunilla Eriksson, Susanne Guidetti

**Affiliations:** 1 Department of Neurobiology, Care Sciences and Society, Karolinska Institutet, Stockholm, Sweden; 2 Uganda Allied Health Examinations Board, Kampala, Uganda; 3 Butabika National Referral Mental Hospital, Kampala, Uganda; 4 Uganda Institute of Allied Health and Management Sciences - Mulago, Kampala, Uganda; 5 Women’s Health and Allied Health Professionals Theme, Karolinska University Hospital, Stockholm, Sweden

**Keywords:** Non-communicable diseases, Activities of Daily Living, Rehabilitation, Occupational therapy, Africa, Rural, Family, Caregiver support.

## Abstract

**Background:**

The global burden of stroke is increasing and persons with low socioeconomic status are among those worst affected. In Uganda, stroke is estimated to be the sixth highest ranking cause of death. The Ugandan healthcare system is reported to be inequitable, where poorer populations often live in rural areas with long distances to health care. Stroke rehabilitation is often scarce, with less financial and human resources. The aim of this study was to explore and describe the consequences of stroke in daily activities in everyday life for people in a rural part of Masaka in Uganda.

**Methods:**

Qualitative study design. Fourteen persons who had had stroke and were living in their home environment were interviewed about their experiences of having a stroke and managing their lives after the stroke incident. The interviews were analysed using thematic analysis. In addition, sociodemographic data and level of independence (Barthel Index and Stroke Impact Scale 3.0) was collected to describe participant characteristics.

**Results:**

Most of the participants had major consequences of stroke and described that they were dependent on support for managing their daily activities. Five themes were identified in the analysis: (1) Accepting and adapting to new ways of managing everyday life, (2) Changing roles and hierarchical positions, (3) Depending on caregiver support, (4) Interrupted care due to economic constraints, (5) Stroke leading to losses and losses leading to stroke.

**Conclusions:**

The consequences of stroke on the persons’ daily lives clearly reached beyond the person with stroke, affecting the whole family and their proximate social networks. These consequences included increased burdens on caregivers and a worsened economic situation for all persons affected. Therefore, interventions for stroke management should preferably not only target the individual affected by stroke, but also support the caregivers in the caring and rehabilitation process. Home rehabilitation approaches with a focus on improving health literacy are suggested.

## Background

Stroke is a global burden, reported as the second highest-ranking cause of death, and the incidence of stroke is predicted to increase further [1]. Persons with low socioeconomic status are among those worst affected [2, 3], and the mortality rate after stroke is higher in this group [3]. The burden of stroke in Africa is increasing [4], and in Uganda, stroke is currently estimated to be the sixth highest-ranking cause of death [5].

The Ugandan health care system is reported to be inequitable [6, 7], since poorer populations often live in rural areas with long distances to health care [8]. More than 70% of the Ugandan population lives within an hour’s walking distance of the nearest government health facility [8]. The Ugandan health care system consists of both public and private providers, and is based on a referral system, where the first contact with health care for people living in rural areas is with the village health team, and where pocket money is often needed for further care [8]. Knowledge of stroke, its impact, risk factors and prevention strategies has been reported to be low in Uganda [9–11], especially in rural areas [10]. Other barriers to stroke rehabilitation and stroke prevention strategies in Uganda are the low availability of existing rehabilitation centers with adequate equipment or financial and human resources [12].

The ability to be independent and participate in activities of daily living (ADL) is often severely affected after a stroke due to cognitive and physical impairments, reducing the quality of life in many respects [13, 14]. Due to factors such as disability after stroke, a gap might emerge between activities a person would like to engage in and what they actually do [13]. Unmet needs among stroke survivors have been reported, such as health-related needs of re-integration into the community [15]. These needs were mostly physical and cognitive, but also included the need to access more easily understood information material [15].

Rehabilitation interventions with a focus on enabling participation in meaningful activities from a client perspective are important, and such interventions are central for people to manage their daily life again. Significant improvements in ADL from occupational therapy have been found globally [16–19]. Research in a European context shows that the main focus should be on ADL, particularly in the acute phase of rehabilitation, but that there is also a need to focus on recovery of previous social roles when returning home for those who have been hospitalized [20]. However, opportunities for rehabilitation are generally limited in the African context [8, 9, 21]. Despite the high numbers of stroke cases in Uganda, there are almost non-existent opportunities to receive professional rehabilitation [12]. In particular, there is lack of rehabilitation personnel such as occupational therapists and physiotherapists.

It is common in African societies to be taken care of by informal caregivers after a stroke, both while receiving emergency care in hospitals and after discharge, and previous studies have reported a high caregiver burden [22–24]. This burden often leads to worsened mental health and wellbeing for the caregivers [23, 25, 26]. In addition, the relationship between stroke survivors and their caregivers can be affected negatively [27]. Female caregivers are overburdened [24], and the strongest determinants for overburdening are the dependency level of the person with stroke and the duration of caregiving [28].

An unmet need of information and support in the care for the stroke survivors among caregivers has been reported from different parts of the world [15]. Social support could lessen the caregiver burden, although varied results have been reported for such interventions [29]. Perceived social support has been shown to lessen the subjective burden to a higher extent, compared with the actual social support received. As such, perceived social support is a good predictor of lessened caregiver burden [30]. Limited social support and stigmatisation could also be obstructing the prevention and rehabilitation of persons with stroke [12].

There are some studies on the impact of stroke and its consequences for people living in sub-Saharan Africa [10, 11, 19, 24, 31], including studies conducted in an urban area in Uganda, the capital city Kampala [19, 31]. However, more research on the consequences of stroke is needed, especially among people living in rural areas where the knowledge of causes and potential treatments have been found scarce [9, 11]. This is important since approximately 84% of the Ugandan population reside in rural parts of the country [32]. Therefore, knowledge is needed as a basis for developing care and rehabilitation interventions also for people living in a rural area.

The aim of this study was to explore and describe the consequences of stroke in daily activities in everyday life for people in a rural part of Masaka in Uganda.

## Methods

Qualitative interviews were conducted with 14 persons who had had a stroke and were living in their home environment. In addition, structured questionnaires, including established assessment instruments, were used to describe characteristics of the study sample. The study is reported in accordance with the consolidated criteria for reporting qualitative studies (COREQ) [33].

### Setting and recruitment

The study was conducted in Masaka, a district located 120 km south of Kampala, the capital of Uganda. The geographical area for this study was selected based on the opportunity to reach persons with experiences of living in a more rural setting, often with lower access to health facilities.

The participants were recruited through the physiotherapy department at Masaka Regional Referral Hospital, which coordinates rehabilitation services in Greater Masaka. Greater Masaka includes other government and private hospitals within and the districts surrounding Masaka City. In addition, the General Population Cohort (GPC) was used for recruitment. The GPC is a community-based open cohort study of residents of neighboring villages within one half of a sub-county, located about 40 km from the shores of Lake Victoria, currently including approximately 22,000 people.

To select participants, purposive sampling was used based on the inclusion criteria: (1) stroke diagnosis confirmed by computed tomography scan or by clinical symptoms, (2) no psychiatric diagnosis, (3) able to understand and respond to instructions in English or the Luganda language.

### Data collection

The main data collection consisted of 14 semi-structured interviews conducted by two Ugandan research assistants knowledgeable in the local language and the culture. The interview guide was developed together with research assistants from GPC, knowledgeable of the living conditions in the geographical area, to adjust the questions accordingly. The interview guide contained questions about the participants’ and caregivers’ experiences of the stroke onset, the recovery process and the current situation, including contact with health care, as well as questions about the participants’ lives before the onset of stroke. All interviews were conducted in Luganda, transcribed verbatim, and translated to English after transcription. The interviews lasted between 55 min and 1 h 40 min. Observations were conducted and fieldnotes taken when visiting participants in their home environment. One of the research assistants wrote summary texts about the meetings with the participants after conducting interviews. and these were also added to the analysis.

Caregivers (i.e., a spouse, daughter, son, neighbor, or friend) were present during most of the interviews and sometimes supported the stroke survivors in recalling their experiences, since it is common in Uganda to live closely together with extended family. Therefore, it was very natural and unavoidable that they were a part of the interview. Almost all interviews ended with questions directed to the caregivers. The questions to caregivers concerned both how the situation after stroke affected them, the stroke survivors, and other people around them. The view on the situation from the participants’ and the family members’ standpoints were however carefully separated during the analysis.

In addition, sociodemographic data was collected as information on stroke severity by Barthel Index [34], and perceived impact of stroke by the Stroke Impact Scale 3.0 - Uganda version) [35].The Barthel Index (BI) includes 10 self-care and mobility activities: feeding, bathing, grooming, dressing, bowel control, toileting, transferring from chair to bed and back, walking on a level surface, as well as ascending and descending stairs. The total score ranges from 0 to 100, and stroke severity is categorized as < 15 = severe, 15–49 = moderate, and 50–100 = mild stroke [36, 37].

The Stroke Impact Scale (SIS) is a self-report questionnaire comprising 59 items that assess the perceived impact of stroke in eight different domains: strength, memory and thinking, emotion, communication, ADL/instrumental ADL (IADL), mobility, hand function and participation. The aggregated score ranges from 0 to 100, where a higher score indicates lower perceived impact of stroke. In addition, a visual analog scale ranging from 0 (no recovery) to 100 (full recovery) assesses general perceived recovery since the onset of stroke [38]. The SIS has been reported to be reliable, valid, and sensitive to change [39]. A culturally adapted and tested version of the SIS 3.0 [35], available in both Luganda and English, was used in this study.

### Participants

The age of the participants was between 42 and 85 years, the majority were female (11/14), and the mean age at stroke onset was 67 years. Results from the BI showed that most participants had a mild stroke. The characteristics of the stroke survivors are presented in the table below (Table 1).

**Table 1:** Participant characteristics of 14 stroke survivors

ID	Age	Gender	Marital status	Years since stroke onset	Stroke severity (BI)*	House-hold members	SIS-domain Strength	SIS-domain Memory	SIS-domain Emotion	SIS-domain Commu- nication	SIS-domain ADL/ IADL	SIS-domain Mobi-lity	SIS-domain Hand function	SIS-domain Parti-cipation	SIS** Stroke recovery
1	42	Female	Married	10	mild	5	31.3	100	63.9	100	85	63.9	0	53.1	70
2	70	Female	Widow	13	mild	2	25	78.6	55.6	89.3	70	50	10	43.8	70
3	58	Male	Divorced	2	mild	11	75	89.3	77.8	85.7	87.5	94.4	100	71.9	10
4	72	Female	Married		mild	6	25	89.3	55.6	100	32.5	44.4	20	18.8	10
5	85	Male	Married	3	moderate	5	0	10.7	50	17.9	22.5	8.3	0	3.1	30
6	60	Male	Separated	4	mild	6	37.5	75	55.6	82.1	70	27.8	65	43.6	30
7	72	Female	Widow	7	mild	5	62.5	89.3	55.6	92.9	85	86.1	70	34.4	50
8	53	Female	Divorced	1	-	2	-	-	-	-	-	-	-	-	-
9	76	Female	Divorced	0	mild	5	62.5	78.6	52.8	75	50	55.6	81.3	56.3	60
10	68	Female	Divorced	5	mild	5	56.3	39.3	52.8	32.1	40	83.3	20	18.8	50
11	60	Female	Not married	5	mild	5	18.8	75	66.7	96.4	52.5	13.9	20	28.6	30
12	60	Female	Married	0	moderate	6	12.5	87.5	38.9	78.6	17.5	8.3	12.5	31.3	50
13	55	Female	Separated	3	mild	8	50	42.7	36.1	42.9	20	30.6	5	28.1	40
14	77	Male	Married	1	mild	9	50	71.4	63.9	75	10	8.3	0	34.4	14

*Stroke severity categorized based on the Barthel Index (BI) scores, < 15 = severe, 15–49 = moderate, 50– 100 = mild (Govan L, Langhorne P, Weir CJ. Categorizing stroke prognosis using different stroke scales. Stroke 2009;40:3396–9.).

** Stroke Impact Scale (SIS) recovery: 0 (No recovery) – 100 (Full recovery).

### Analysis

The analysis of transcribed interviews was inspired by thematic analysis described by Braun and Clark e [40, 41]. The interviews were analyzed by LT, GE and SG. Initially, the transcripts were read several times to immerse into the data. Thereafter, themes were identified inductively based on selected meaning units from the coding process (LT). Startup meetings online on Zoom were arranged between LT and one of the Ugandan research assistants (SK), who also conducted the majority of the interviews. This to discuss the interviews to avoid contextual misunderstandings. Most of the transcribed texts were also read by two researchers (LT and SG) and the content was discussed between them in order to reach a common understanding. In addition, discussions were held between the co-authors. In this process new themes were developed and some of the initial themes were revised. For clarifications, the research assistant SK was contacted at several timepoints. The interview data included in the analysis was considered to have enough information power, due to the narrow aim and dense specificity of the study sample and the quality of the dialogue, for conducting a rigorous analysis [42].

## Results

A common pattern in the participants’ narratives was the process from independency to dependency and back towards independency again. In that journey, the stroke appeared as a sudden event and destroyed the participant’s former life by the loss of roles important to their identities. Their former daily activities were no longer possible to do in the same way they did them before. This was often due to paralysis leading to impaired hand and arm functions and problems with walking Communication problems were common due to aphasia and cognitive impairment. Former roles were lost and replaced by new ones. It seemed like for many of the participants, striving for independency was a driving force in their progress towards recovery.

Five themes were identified: (1) Accepting and adapting to new ways of managing everyday life, (2) Changing roles and hierarchical positions, (3) Depending on caregiver support, (4) Interrupted care due to economic constraints, and (5) Stroke leading to losses and losses leading to stroke.

The themes are presented below using quotes from the participants to illustrate the findings.

### Theme 1. Accepting and adapting to new ways of managing everyday life

The consequences of stroke were evident in activities of everyday life for all participants, where different ways of handling their perceived impact of stroke were noted. One way was through acceptance of the circumstances, which had been achieved by finding new strategies to manage everyday life. The participants described that they both needed to adapt themselves to new roles and positions and adapt in relation to the environment i.e., the situation of being stuck at home because of immobility. In striving for independency, the participants described how they needed to develop their own strategies to be able to manage their daily activities independently. The following quote illustrates one example of a strategy to wash oneself:

*I organize a cloth so that I scrub myself with this hand of mine in this area like this with a sponge. Then after scrubbing, then make the cloth like this, this one is the one which helps me to fetch water the way you do this. Or sometimes I do like this. (ID: 7)*

Another strategy to adapt to the circumstances was to use handmade assistive aids to be able to manage daily activities - for example, to use a walking stick to be able to fetch water when there were no relatives or neighbors who could help. Adaptation to the new situation in everyday life was apparent when participants got used to their conditions - for example, by accepting unavoidable dependency. The following quote is from a woman showing an acceptance of being dependent on others help for certain activities:

*I’m used to it. Yes, I mean I’m used… So now I call them and I’m like please come come its time… and they come. And they hold onto me. And I wake up. Umm I’m used to it. I don’t have any more problem. (ID: 4)*

Most of the participants described that they, after some time, had accepted their health status as permanent but nevertheless their pursued independency could be seen. After a period of denying the situation of the impact of stroke, the process often led to an acceptance of the new circumstances. One participant described how the process had developed from feeling sorry for oneself to a decision on the importance of doing what he could despite limitations, and to work at least to the extent that was possible:

*I’ve seen the situation is permanent…There is a period I pity myself in life, it’s inevitable. Then I say to myself that even if I pity myself, it came… it’s not going back, I am not now expecting that I will go back to the way I was before. And then I got used to it so that I can work within it, within it. When I get something to do here…I do it for myself so that I know that I earn something, a shilling. (ID: 7)*

Some participants expressed a feeling of being stigmatised, by persons in their proximate social network and by people they did not know from before. Some participants got offended by other people’s reactions when they felt sorry for them and it was experienced as shameful to be in this situation. To sit in a wheelchair was also considered stigmatising. The following quote is from a woman who thought that some people just visited her to check her status and to gossip about her:

*They check on me…They back-bite me. But they don’t give me help. No, the person only makes effort to come to see which situation you’re in… then that’s what they gossip around. (ID: 4)*

In line with the previous quote confirming the stigmatisation, another woman explained that she got offended earlier but nowadays she was used to her health status and had accepted it:

*Yes, when people came uh… look at her… that lady used to be very beautiful huh there I would cry. (ID: 1)*

A strong belief in God could be noted both among the participants with stroke and their caregivers, and progress was seen as being in the hands of God. An acceptance of the situation related to God’s will appeared also common in the participants’ narratives. Several examples were reported of prayers for recovery:

*Uhh it involves a lot of things indeed that’s why I ask God quite enough to deliver me and make this hand heal quickly. (ID: 14)*

The rehabilitation process could be thought as dependent on decisions from God, which seemed beyond the person’s own control, requiring an acceptance of one’s health status. The following example illustrates one caregiver’s acceptance of the situation of having a husband with stroke:

*Yes, as you know God is so powerful… Then he got back to understanding and began talking, but we could not comprehend what he said … and for me I was just accepting…since then he got paralysed, the legs got contractures, uh, the other hand died there, and it’s stuck there.(ID: 5, wife/caregiver)*

Many examples confirm that the participants had learned to live with their shortcomings. They had adapted to the situation and had accepted their dependence on others. While some participants complained about burdening others, many of them were not as bothered as they were initially. A progress in health status from the initial phase was often described, both through natural recovery achieved with time and through the strategies developed.

### Theme 2. Changing roles and hierarchical positions

The changes of roles due to the participants’ stroke were apparent. For example, one person experienced a transition from being a wife to becoming a daughter, when the situation forced her to move back to her mother and to be taken care of just like a child again. The same person was earlier a singer in a band of musicians, and worked in agriculture, but was suddenly not able to do any of her former activities, losing all roles related to her identity.

The new roles could also lead to changes of status in the family. A person who had been taking care of the others within the family could suddenly be in the position of needing care, and the ability and power of being in charge of the family and the decision making was lost. A daughter to a man who had a stroke explained the change of roles and responsibilities they had experienced as follows:

*On our life there is a very big difference, ever since father got stroke there is a change because there are some things that father used to do at home when for us we weren’t here, he could do many of them even taking care of his wife, our mother, he could bring her what to eat which is impossible right now, father is now incapable of giving what helping at home, except looking after him, yet back then he was the one who was taking care of all of us.(ID: 14, sister/caregiver)*

However, there were also examples when the hierarchical positions within the family remained, despite that the head of the family was immobilised due to paralysis caused by stroke. It could for example be possible to adapt the work tasks for the person having had a stroke so that s/he could continue to work to some extent. The quote below illustrates a way to maintain the connection to working life by keeping contact with the workers and manage the role of supervisor, instead of doing the hands-on work that he did himself before the stroke:

*Except when I do… when it don’t happen I go there and I supervise the workers. I have done like that… (ID: 6)*

As shown above, many examples of losing former roles and meaningful activities were found. In addition, activities such as gardening and cooking, which were important to the participants’ identity, were lost. Participants were often expressing a desire of wanting to engage in former activities and occupations, often related to work that gained income, and to thereby contribute to taking care of the household. One example is a woman who chose to start a small-scale business close to a school, where she was selling small items such as pieces of soap, sugar, sugarcane, and other sweets for children to buy. She had help from a supportive friend who purchased the things needed from a market and baked pancakes for her to sell.

### Theme 3. Depending on caregiver support

The need for and the importance of caregiver support was obvious throughout all interviews. The persons who had had a stroke needed both emotional support and support in daily activities to manage their lives, and all of them were dependent on having people around. It was most common to be taken care of by family members and relatives, but also friends and other persons from proximate social networks could be involved in care. Children were also often involved in care - in rare cases, external persons such as maids were paid for assisting.

The provided help was necessary for managing daily living, and it was acknowledged and highly appreciated by the persons with stroke. The need of social support was also central for well-being, since loneliness was expressed as causing depression. However, there were also examples of participants feeling bad because of all the help needed from and provided by relatives, and they could at times be moved from living with one caregiver to another due to limited resources and caregiver burden. To be taken care of was at times expressed as shameful. Several examples could be found that the persons with stroke felt bad and were ashamed when their children and grandchildren had to take care of their bodily wastes. One participant expressed a concern about the risk for her daughters to lose their possibility of marriage due to caring for her.

Caregiver burden was also expressed by the caregivers. This quote by a caregiver, who had been taking care of her sister since she had a stroke. illustrates the lack of other options and the responsibility for taking care of your relatives:

*Because she is of the same blood, then where can I dump her? (ID:9, sister/caregiver)*

The new circumstances could also lead to reorganisation of living conditions. The quote below illustrates a process of coming to an understanding of the benefits of living together to help each other when experiencing limitations in everyday life:

*Um, until I got used [to it] and I saw those that prepared for me my food, by then my aunt was still [on] the other side… in Ngolwe. So then I reached a consensus and I said to myself that in this sickness of mine…will I be in one place. Then I sold my land there, part of it, and he started building for me a house there. My aunt [on] the other side had some problems too, and I brought here there…she could peel, and we cook on a charcoal stove. (ID: 7)*

The reorganisation of living conditions was necessary for being able to manage everyday activities such as cooking food. Despite both the person with stroke and her aunt having limitations in the ability to perform the tasks required for independent living, the teamwork made the activities possible.

Some caregivers seemed to serve as motivators to training by encouraging exercise and participation in daily activities. However, other examples could be seen showing the opposite. Some participants experienced their caregivers as hindering them from doing activities, where the participation in doing daily activities was seen as unnecessary by caregivers.

### **Theme 4: Interrupted care due to economic constraints**

In general, the participants living in the rural area in this study had low socioeconomic status. They were willing to test everything in terms of treatments available but were often hindered by the high costs. The health care chosen was based on the lowest costs. However, the participants with stroke were often discharged from hospitals due to lack of money. Also, the cost of transportation could prevent hospital visits. Several examples were found on interrupted care, where the participants could not continue with the recommended medications due to economic constraints. The quote below illustrates a situation where it was not possible to continue treatment:

*It helped me there on a small basis. Because I didn’t… I finished something like three weeks. Because of money they discharged us. We begged them but they had insisted on me and I told them where shall I get the money…? (ID: 7)*

The participants had often been offered medication- both traditional herbal medicines and Western medicines were frequently used. High blood pressure was recognised as a risk factor for stroke, and participants reported that medication for this purpose was needed. In addition, many participants suffered from comorbidity in terms of (for example) prediabetes or diabetes. The traditional medicines were more affordable and therefore often chosen, although not all people around them trusted the traditional medications, as shown in the following quote:

*People, in my initial stages…they used to tell me those things… that it’s witchcraft. But because of my poor wallet. (ID: 7)*

Medication was also at times used as a prevention strategy when it could be afforded, which can be interpreted as an awareness of potential risks of getting affected by hidden diseases such as prediabetes:

*Participant: [Laughs] we always drunk the medicine saying, uh, hopefully the diseases might not be in the body… And we drunk some…*

*Interviewer: Uhh, which kind of disease were you frightened about?*

*Participant: Stroke, diabetes, those what and the rest of the diseases.*

*(ID: 9)*

A clear lack of opportunities for professional rehabilitation was expressed both by the participants and caregivers. This was due to both lack of money and availability for people living in rural areas. However, an awareness of the benefit of training could be seen among some participants and their caregivers, where they mostly had received advice for training from a doctor or nurse. One participant talked about a strategy of tying a rope between two trees to use as support for walking, but this exercise had not yet been practiced. Training through activities is illustrated in the quote below, where the participant clearly described how she trained the affected hand by practising when doing daily activities:

*…I was there feeling paralysed, even the fingers.. .okay, the fingers got an attack, and they did like this… But I remained with these ones. I was there okay holding things like that, I was forcing it, um, even now there reaches a time and it refuses when I force it to eat. (ID: 2)*

The stays at hospital and medication received were often interrupted due to lack of money. Some of the hospital care was supposed to be free of charge, but this was often not the case. Also, transportation between home and hospital was hindered due to costs. Although the rehabilitation opportunities were scarce, the economic constraints seemed to be the main reason for not continuing the treatments available. Despite lack of rehabilitation, medication and interrupted care due to high costs, a recovery process was described. Many of the participants strived for independency in their recovery process, while other accepted their situation and took a more passive rehabilitation approach.

### Theme 5. Stroke leading to losses and losses leading to stroke

Many participants reported lost income due to the stroke that in turn affected their possibilities for potential treatments. One participant stated that she needed to sell the last harvest to pay the hospital bill and that she then couldn’t afford more care. The stroke could often lead to a loss of income and as a consequence, property needed to be sold in order to afford what was needed. One participant lost her property while staying at her sister’s house, since she needed the sister’s care. During the time she lived with her sister, the sister’s son sold the participant’s land without permission from her. Since she was dependent on the support of her sister, she decided to keep quiet.

Also, caregivers lost their possibilities to earn money to the extent they did before because they needed to be present with their relative affected by stroke. There were also examples of family members and relatives worsening the situation and causing stress for the person who had had a stroke - for example, by selling off property, leading to a worsened economic situation. A caregiver who was present during one of the interviews explained it like this:

*Lady: Hmm, yes, for sure, it seems the children came, the children of the husband, should say, started selling portions of land.*

*Interviewer: Uh-huh, so that person goes into a state of stress*.

*Lady: Yes so, that’s what the neighbor told us, that they went on dividing portions of the land as they sold them, and I should say they left her with only a portion of where the house was situated.*

*(ID: 13, lady/caregiver)*

The lack of money could also affect the relationship to the people close to you. One caregiver described this situation as follows:

*Yeah, she used to earn some little from her farming. Before she was used to having her money in the pocket, now that also has a way, it affects her (…) if she doesn’t do that then it will affect her …and she buys her grandchildren something. If she doesn’t do that then it will affect her.*

*(ID: 12, woman/caregiver)*

The income losses lead to prioritisation of expenses and unwanted changes in diet, where the foods that the persons who had had a stroke used to enjoy were no longer an option. Another example was that a couple separated after a long-lasting marriage, which led to further income losses for the household. The same participant was a farmer before she had a stroke some years earlier. She told about her losses in not being able to keep up the farming:

*All my things have perished, they have died out. I don’t have even where I could start from now. All my things have perished. (ID: 13)*

Losses of income for both stroke survivors and their caregivers were reported and due to the income losses property was lost. In almost all cases, the living conditions had markedly worsened in families where a family member had been affected by a stroke, causing a negative impact on the families’ overall wellbeing.

## Discussion

This study focused on the consequences of stroke in people who live in a rural area, given that previous studies mainly explored the situation among people with stroke in urban areas. The participants perceived stroke as having substantial consequences on their daily lives.

The participants strived to adapt to new roles and positions, and partly formed a new self-identity by doing so. Recovery from stroke often seemed to lead to an acceptance of the new situation of not being independent to the same extent as earlier. This included adaptations and changes of former roles, finding new forms for one’s self-identity. These findings confirm those of a previous study based on a similar study population, where loss of self-identity after stroke was reported [21]. The process of acceptance could include an initial feeling of being ashamed and wanting to hide from other peoples’ reactions and stigmatisation, but also subsequent feelings of being less affected by others’ reactions. The participants’ strong belief in God, and that it was God’s will that they had suffered a stroke, possibly contributed to the acceptance of their situation.

Our findings on the importance of participation in everyday activities and of striving for independence to reach a sense of satisfaction are in line with those of a previous study [14]. The progress in performing daily activities after having a stroke has been expressed as happening slowly through repetition [14]. Although all participants described an initial progress in health status from the stroke onset, it is likely that their recovery process would have progressed further with more accessible rehabilitation, including performance of daily activities [16–19].

The dependence on caregivers was evident and confirms the findings from our previous study with participants from a more urban setting [23]. The support from caregivers was central, and a significant part of managing daily life for most of the participants. Nevertheless, some caregivers seemed to be more rehabilitation-oriented and encouraged participation in activities, while others were more disturbed by the involvement of the persons affected by stroke and actively hindered their participation, as previously found in the Ugandan context [23]. A raised awareness of the benefits of engagement in activities seemed to be important for managing daily activities and could improve levels of independence. Although the benefits of involvement in activities need to be further investigated in this context, interventions focusing on caregivers have the potential to promote rehabilitation through activities [23].

Despite that the caring of persons with stroke was expressed as burdensome by caregivers, in terms of workload and loss of income, there was a natural commitment to taking on the caregiver role. It was also apparent that there were no other options available to caregivers than to adapt and adjust to the life circumstances in their situation. The result indicated that it was most often female relatives who took the greatest responsibility for caregiving, which is in line with findings in previous studies [23, 24]. The families expressed being highly burdened by the stroke, both economically and with an added workload. Caregiver burden has frequently been reported by caregivers to people with stroke in African contexts, based on the same kind of experiences as those reported in our study sample [22, 24, 43, 44]. It might be considered that leaving one’s home to take a caregiver role, which was common in this rural context, would generate even more burden.

In line with previous studies, this study confirmed that the burden on caregivers affects their mental wellbeing [23, 25, 26]. Since social support has been found to decrease mental health problems for caregivers to persons with stroke [25], peer support emerges as a suitable intervention approach to implement for caregivers. Peer support involves caregivers meeting each other to share experiences and strategies to manage caring and everyday life, together with the persons affected by stroke. The dependence and involvement of the caregivers in everyday activities highlight the need of expanding the focus on client-centered practice to reach beyond the person-centeredness to a more family-based practice.

Accessible stroke information has been requested by stroke survivors and caregivers [12] and is urgently needed to raise the knowledge and awareness of stroke and how to manage its consequences in daily life to support the families burdened by caring. Knowledge of stroke, its risk factors, prevention and rehabilitation strategies have been reported as low in Uganda [9–12], which also might have been the case in this study. Thereby, to increase the chances to lessen stroke incidence, interventions to increase health literacy are needed [47]. In the ongoing mass health awareness campaign for the general population in Uganda [12] stroke awareness messages have been recommended to be included.

For all participants, the stroke had led to a loss of income that affected the whole family, leading to interrupted care and inability to buy medications. Although the basic care at Masaka Hospital is free of charge, most examinations and treatments require out-of-pocket money. The assistive aids available are costly, and it was therefore common to make walking sticks from coffee tree. Wheelchairs are mostly imported from abroad, but hardly affordable for people living in rural areas. Our findings also confirmed transportation costs as an obstacle for care seeking, and for access to health care and medication [8]. Poor populations often live in rural areas, and our findings thus confirm that an incidence of stroke affects people of lower socioeconomic status to a greater extent, leading to poorer health outcomes [2, 3].

It is evident that access to rehabilitation in Uganda is limited, specifically in rural areas. However, apart from confirming transport costs as an obstacle to care and medication [8], the participants from rural areas described the recovery process after stroke similarly to how recovery is described by people in more urban settings [23, 24, 31]. The dependence on caregivers was found to be similar in rural and in urban settings, although it seems to be more common that the caregivers in rural areas move into the stroke survivors’ homes, instead of the opposite, where the stroke survivor moves into the caregivers’ homes. To stay in the home environment could potentially encourage involvement in familiar activities that could benefit the recovery process compared to moving into another person’s home, where a more passive role could be natural. We have in our previous studies found that familiar contexts and objects could support the doing of activity, as well as the discovery of the new body and self [45]. Thus, by being in a familiar environment contributed to new meaningful experiences which in turn contributed to the recovery process.

A limitation in the study was that the interviews were conducted in Luganda, translated to English and in a later stage analysed by researchers from a non-African context. However, the research team included co-authors contributing to the cultural understanding and knowledge of the study participants which minimised the potential risk of bias. In addition, data is missing on characteristics for one of the participants.

In summary, the clinical implications we suggest are that support and interventions, should target not only the stroke survivors. As in this African rural context a lot of the responsibilities for the rehabilitation and daily life are required from the caregivers/family members they also need information and support regarding the whole situation. A specific focus on health literacy, self-management approaches and tools for home rehabilitation have been identified to improve performance in daily activities and participation in occupations of persons after a stroke.

## Conclusions

The stroke survivors in this study perceived a high impact of stroke, and they were dependent on support from their caregivers for managing their daily activities. The consequences of stroke clearly reached beyond the person with stroke to affect their families and their proximate social network, both through the caregiver burden and worsened economic situations. The rehabilitation intervention should target the person affected by stroke, but also support the caregivers in the caring and rehabilitation process. Home rehabilitation approaches with a focus on improving health literacy are suggested.

## Data Availability

The data and materials will be available on request from the Division of Occupational Therapy, Department of Neurobiology, Care Sciences and Society, Karolinska Institutet. E-mail: Linda.timm@ki.se.

## Abbreviations

ADL: Activities of daily living
BI: Barthel Index
COREQ: Consolidated criteria for reporting qualitative studies
GPC: General Population Cohort
SIS: Stroke Impact Scale
